# Effect of cold provocation on vessel density in eyes with primary open angle glaucoma: An optical coherence tomography angiography study

**DOI:** 10.1038/s41598-019-45386-7

**Published:** 2019-06-28

**Authors:** Wei-Yi Chou, Catherine Jui-Ling Liu, Mei-Ju Chen, Shih-Hwa Chiou, Wei-Ta Chen, Yu-Chieh Ko

**Affiliations:** 1 Department of Ophthalmology, Taipei Veterans General Hospital, Taipei, Taiwan; 2 Faculty of Medicine, National Yang-Ming University School of Medicine, Taipei, Taiwan; 3 Department of Medical Research, Taipei Veterans General Hospital, Taipei, Taiwan; 4 Neurological Institute, Taipei Veterans General Hospital, Taipei, Taiwan; 5 Institute of Clinical Medicine, School of Medicine, National Yang-Ming University, Taipei, Taiwan

**Keywords:** Optic nerve diseases, Blood flow

## Abstract

The cold pressor test (CPT) induces a cardiovascular response, which may affect ocular blood flow and neuronal function. This study assessed whether optical coherence tomography angiography (OCT-A) can be used to evaluate CPT-induced changes in healthy eyes and in eyes with primary open-angle glaucoma (POAG). Twenty-two healthy subjects and 23 subjects with POAG and retinal fibre layer defects in only one hemifield were included in this study. The CPT was performed by submerging a subject’s hand in cold water (0–4 °C) for 1 minute. The results showed that baseline peripapillary and macular vessel density (VD) measurements were significantly lower in subjects with POAG than in controls (all P < 0.05). Post-CPT VD measurements did not significantly differ from baseline in either healthy or glaucomatous eyes. Additionally, CPT-induced changes in VD did not differ among normal eyes, damaged and undamaged glaucomatous hemifields. Changes in VD were also not significantly influenced by self-reported history of cold extremities. In conclusion, the CPT does not induce significant VD changes, as measured by OCT-A, in the peripapillary or macular areas of either healthy eyes or eyes with POAG. The VD, an all-or-nothing flow measure, may not be sensitive enough for evaluating cold-induced ocular haemodynamic changes.

## Introduction

Glaucoma is the leading cause of irreversible blindness worldwide^1^. Elevated intraocular pressure (IOP) is a major risk factor for glaucoma development and progression. Despite the reduction of IOP, disease progression is still observed in a number of patients^2^. Therefore, other pathogenetic mechanisms for glaucoma, such as vascular dysregulation^3–6^, have been proposed. Dysregulation of ocular blood flow can lead to astrocyte activation, compromised mitochondrial function in retinal ganglion cell axons, and apoptotic ganglion cell death^7^. Furthermore, systemic vascular dysregulation has been found in some primary open-angle glaucoma (POAG) patients, including nailfold capillary abnormalities and impaired flow- and medication-mediated vasodilatation in the brachial artery^8–10^. Accordingly, stress tests used for evaluation of autonomic and endothelial dysfunction in systemic vasculature are used in glaucoma patients to evaluate the vascular component of glaucoma and to identify patients at risk^5,11–13^.

The cold pressor test (CPT) has been used to evaluate the role of vascular dysregulation in POAG in several studies^5,11,12,14^. Constriction of nailfold capillaries and delayed recovery of skin temperature following CPT were related to visual field deficit and progression in POAG patients^12,14^. The CPT was initially used to assess cardiovascular reactivity^15,16^ and has been a safe and simple stimulus for evaluation of the risk of cardiovascular disease, as well as coronary endothelial function^17–19^. CPT is conducted by immersing one hand into iced water (approximately 1 °C) for 1–5 minutes^15,16,20,21^. The cold stimulus elicits arterial vasoconstriction and a blood pressure (BP) increase via sympathetic autonomic nervous system activation^22^. In glaucoma patients, CPT induced an abnormal increase in plasma endothelin-1, a strong vasoconstrictor^11^. Gherghel *et al*.^5^ found that the CPT causes a decrease in blood flow velocity at the temporal neuroretinal rim in eyes with POAG, but not in normal controls. Guthauser and Flammer^12^ demonstrated that visual field (VF) sensitivity was lowered by the CPT and that this change was associated with a history of cold hands and cessation of blood flow in nailfold capillaries after CPT. In patients with normal tension glaucoma, delayed skin temperature recovery following CPT was associated with disease progression^14^. These findings suggest that CPT may be a useful vasospastic stimulus to evaluate ocular haemodynamic responses to stress. If the stress response of the ocular vasculature can be precisely and reproducibly recorded, these findings may lead to further understanding of the vascular component in glaucoma pathogenesis and its association with clinical findings, such as disease progression.

The introduction of optical coherence tomography angiography (OCT-A) allows the peripapillary and macular microvasculature to be rapidly and noninvasively visualized with good reproducibility^23^. As a result, glaucoma research that utilizes OCT-A has rapidly increased in recent years^24,25^. However, the role of OCT-A in evaluating dynamic ocular blood flow in eyes with glaucoma has not been established. Measurements obtained with OCT-A have been used to demonstrate a decrease in peripapillary vessel density (VD) following hyperoxia in healthy participants^26^ and an increase in peripapillary VD following IOP reduction in glaucomatous eyes^27^. Therefore, we are interested to know whether OCT-A can aid ophthalmologists in evaluating the vascular dysregulation in patients with POAG. The current study assessed whether or not OCT-A can be used to evaluate haemodynamic changes in the optic nerve head (ONH) and macula following CPT in healthy eyes and eyes with POAG.

## Results

### Repeatability of optical coherence tomography angiography measurements

The intraclass correlation coefficient (ICC) for two consecutive baseline scans was 0.95 [95% confidence intervals (CI): 0.87–0.98] for whole ONH scans and 0.96 (95% CI: 0.91–0.99) for whole macular scans. The coefficient of variation (CV) of repeated baseline measurements was 2.81% for whole ONH scans and 4.08% for whole macular scans. The inter-session CV of baseline and post-CPT VD measurements was 4.41% and 7.29% for whole ONH and macular images, respectively.

### Baseline characteristics and the effect of the cold pressor test on systemic cardiovascular parameters

The demographic and ocular characteristics of included subjects are summarised in Table 1. Glaucoma patients were significantly older (P = 0.03) and more likely to have hypertension and hyperlipidaemia than the control group (P = 0.02 and P = 0.08, respectively). The proportion of participants with self-reported cold extremities was similar in both study groups. The majority of POAG subjects (78.3%) had pre-treatment IOP less than 21 mmHg. All POAG subjects were treated with topical IOP lowering medications, and most of them (56.5%) had more than one medication. The most commonly used eyedrops were prostaglandin analogs (87.0%), followed by beta-blockers (43.5%), carbonic anhydrase inhibitors (43.5%), and alpha-adrenergic agonists (4.3%).

**Table 1:** Demographic and ocular characteristics of included subjects.

	Control group (n = 22)^†^	POAG group (n = 23)^†^	P
Age (years)	43.2 ± 9.6	50.0 ± 9.9	0.030
Male, % (n)	18.2 (4)	43.5 (10)	0.067
**Self-reported history of:**			
Hypertension, %(n)	0 (0)	30.4 (7)	0.020
Diabetes mellitus, %(n)	0 (0)	0 (0)	NA
Hyperlipidaemia, %(n)	0 (0)	17.4 (4)	0.080
Cold extremities, %(n)	40.9 (9)	56.5 (13)	0.295
Axial length (mm)	24.7 ± 1.0	25.3 ± 1.3	0.134
Central corneal thickness (µm)	536.2 ± 32.7	553.8 ± 36.6	0.109
Visual field MD (dB)	NA	−2.8 ± 3.3	NA
RNFL defect in the superior hemifield, % (n)	NA	26.1 (6)	NA
Pre-CPT IOP (mmHg)	15.9 ± 2.7	14.0 ± 2.7	0.021
Pre-CPT BP (mmHg) Systolic Diastolic	118.5 ± 16.8 75.0 ± 11.0	120.4 ± 14.4 76.0 ± 11.5	0.706 0.488
Pre-CPT PR (bpm)	78.9 ± 12.5	77.5 ± 15.3	0.893
Post-CPT BP (mmHg) Systolic Diastolic	118.2 ± 16.2 76.4 ± 11.5	123.6 ± 11.3 76.8 ± 10.3	0.439 0.815
Post-CPT PR (bpm)	76.0 ± 10.6	75.3 ± 14.8	0.957

^†^All values are presented as mean ± SD unless otherwise noted.

POAG, primary open-angle glaucoma; MD, mean deviation; dB, decibel; RNFL, retinal nerve fibre layer; CPT, cold pressor test; IOP, intraocular pressure; BP, blood pressure; PR, pulse rate; NA, not applicable.

Glaucoma and healthy subjects had similar baseline cardiovascular parameters. The CPT did not induce a momentous change in either BP or pulse rate (PR), except for an increase in systolic BP after cold provocation in the POAG group (120.4 ± 14.4 mmHg vs. 123.6 ± 11.3 mmHg, p = 0.04).

### Effects of the cold pressor test on vessel density

The OCT-A measurements obtained before and after the CPT are summarised in Table 2. Age was not correlated with baseline ONH or macular VD measurements in healthy eyes (Pearson correlation coefficient 0.054 and −0.066 for whole image ONH and macular VD, respectively). However, all baseline parameters, including ONH and macular VD, were significantly lower in POAG subjects than in healthy controls (all P < 0.05). The CPT did not induce significant changes in VD in either group (all P > 0.05). Furthermore, CPT-induced VD changes did not differ among hemifields in healthy eyes, damaged glaucomatous hemifields, and undamaged glaucomatous hemifields (Table 3).

**Table 2:** Baseline and post-cold pressor test vessel density measurements.

	Control (n = 22)		POAG (n = 23)		P*	P**	P***
	Baseline	After CPT	Baseline	After CPT			
**Peripapillary vessel density (%)**							
Whole image	53.3 ± 5.6	53.7 ± 5.2	46.4 ± 4.5	46.6 ± 4.5	<0.001	0.592	0.879
Circumpapillary	58.3 ± 5.1	58.5 ± 4.8	51.7 ± 5.7	51.9 ± 5.4	<0.001	0.958	0.796
Nasal	54.6 ± 5.6	55.4 ± 5.2	48.5 ± 6.9	48.2 ± 6.8	0.004	0.355	0.37
Inferior nasal	59.1 ± 5.8	59.2 ± 4.6	53.8 ± 8.1	54.8 ± 7.0	0.021	0.935	0.26
Inferior temporal	62.8 ± 5.6	62.3 ± 5.7	50.5 ± 9.7	49.7 ± 9.5	<0.001	0.237	0.316
Superior temporal	60.6 ± 6.3	60.2 ± 6.0	53.9 ± 8.3	53.7 ± 7.8	0.006	0.426	0.784
Superior nasal	60.2 ± 3.7	59.7 ± 3.7	54.9 ± 6.5	54.5 ± 7.2	0.003	0.399	0.976
Temporal	58.2 ± 6.8	58.7 ± 6.9	52.9 ± 8.0	53.9 ± 7.3	0.016	0.527	0.494
**Macular vessel density (%)**							
Whole image	48.7 ± 7.6	49.0 ± 8.0	40.1 ± 5.5	39.9 ± 5.8	<0.001	0.305	0.627
Fovea	26.1 ± 5.9	26.4 ± 6.7	20.8 ± 5.8	21.1 ± 6.3	0.003	0.414	0.605
Parafovea	51.0 ± 8.1	51.2 ± 8.4	42.2 ± 6.2	42.0 ± 6.5	0.001	0.664	0.605
Superior half	50.8 ± 8.2	51.1 ± 8.4	42.2 ± 5.9	42.1 ± 6.1	0.001	0.741	0.412
Inferior half	51.1 ± 8.0	51.2 ± 8.4	42.1 ± 6.8	41.9 ± 7.0	<0.001	0.543	0.715
Temporal quadrant	50.6 ± 7.1	50.3 ± 8.0	42.1 ± 5.7	41.6 ± 5.9	<0.001	0.768	0.939
Superior quadrant	51.4 ± 8.6	51.9 ± 8.6	42.5 ± 6.8	42.7 ± 6.3	0.001	0.848	0.429
Nasal quadrant	50.1 ± 8.7	50.1 ± 8.9	41.8 ± 6.5	41.7 ± 7.2	0.001	>0.999	0.616
Inferior quadrant	51.8 ± 8.4	52.3 ± 8.4	42.4 ± 7.3	42.1 ± 7.4	<0.001	0.498	0.843

*Comparison of baseline VD in healthy controls and eyes with POAG (Mann-Whitney *U* test).

**Comparison of baseline and post-CPT VD measurements in healthy controls (Wilcoxon signed rank test).

***Comparison of baseline and post-CPT VD measurements in eyes with POAG (Wilcoxon signed rank test).

POAG, primary open-angle glaucoma; CPT, cold pressor test; VD, vessel density.

**Table 3:** Vessel density changes induced by the cold pressor test in healthy and glaucomatous eyes. Measurements made in the undamaged and damaged hemifields in glaucomatous eyes were compared to measurements made in healthy eyes.

	Control (n = 22)	POAG eyes Superior hemifield		POAG eyes Inferior hemifield		P*
		Undamaged (n = 17)	Damaged (n = 6)	Undamaged (n = 6)	Damaged (n = 17)	
Change in peripapillary vessel density (%)						
**Circumpapillary**						
Superior temporal	−0.43 ± 3.76	−0.48 ± 5.07	0.57 ± 5.52	NA	NA	0.953
Superior nasal	−0.46 ± 3.13	−0.50 ± 7.08	−0.09 ± 4.72	NA	NA	0.916
Inferior nasal	0.18 ± 3.70	NA	NA	0.11 ± 5.04	1.31 ± 3.23	0.247
Inferior temporal	−0.49 ± 3.16	NA	NA	−1.29 ± 3.76	−0.68 ± 3.29	0.973
Change in macular vessel density (%)						
**Parafovea**						
Superior half	0.26 ± 3.70	−0.05 ± 5.56	−0.09 ± 1.97	NA	NA	0.716
Superior quadrant	0.47 ± 3.88	0.42 ± 6.23	−0.52 ± 2.18	NA	NA	0.700
Inferior half	0.11 ± 3.68	NA	NA	0.14 ± 1.28	−0.38 ± 6.58	0.927
Inferior quadrant	0.55 ± 4.15	NA	NA	0.83 ± 1.54	−0.76 ± 7.70	0.728

*P-value calculated using the Kruskal-Wallis test.

POAG, primary open-angle glaucoma; NA, not applicable.

Changes in VD following CPT in subjects with self-reported cold extremities did not significantly differ from subjects without self-reported cold extremities (Table 4, all P > 0.05). Additionally, CPT-induced VD changes were not significantly altered when POAG subjects with hypertension or topical beta-blocker use were excluded from analyses.

**Table 4:** Changes in vessel density following the cold pressor test in subjects with and without a self-reported history of cold extremities.

	Control group (n = 22)		P*	POAG group (n = 23)		P*
	Cold extremities			Cold extremities		
	No (n = 13)	Yes (n = 9)		No (n = 10)	Yes (n = 13)	
**Change in peripapillary vessel density (%)** ^**†**^						
Whole image	0.41 ± 2.35	0.48 ± 2.52	0.794	0.95 ± 4.30	−0.33 ± 1.97	0.376
Circumpapillary	0.21 ± 2.49	0.19 ± 3.12	0.556	0.56 ± 4.23	−0.14 ± 2.61	>0.999
**Change in macular vessel density (%)** ^**†**^						
Whole image	0.19 ± 3.47	0.55 ± 2.61	0.464	0.65 ± 5.04	−0.89 ± 3.94	0.131
Fovea	0.54 ± 1.89	0.06 ± 1.43	0.754	0.50 ± 3.77	0.22 ± 2.61	0.648
Parafovea	−0.07 ± 4.06	0.52 ± 3.18	0.422	0.59 ± 5.99	−0.85 ± 4.18	0.208

*P-value calculated using the Mann-Whitney *U* test.

^†^Only representative parameters shown. None of the sectoral parameter comparisons were statistically significant.

POAG, primary open-angle glaucoma.

## Discussion

The present study did not find significant CPT-induced changes in peripapillary or macular VD in either healthy eyes or those with POAG. Additionally, the response to the CPT did not differ between subjects with and without a self-reported history of cold extremities. In agreement with prior studies^24,25,28^, baseline measurements showed that peripapillary and macular VD were attenuated in eyes with POAG (compared to healthy controls).

The CPT did not elicit significant cardiovascular changes. However, it should be noted that all post-CPT BP measurements were made 5 minutes after CPT completion, following all post-CPT OCT-A evaluations. A prior community-based screening study revealed that CPT-induced BP elevations reach their peak 1 minute following CPT, returning to baseline after 4 minutes^17^. In addition, the response to CPT was more obvious and prolonged in patients with hypertension^18^. In our study, the POAG group had several patients with hypertension, and we did observe an increase in systolic BP in POAG patients, but not in normal controls.

Vascular dysregulation has been linked to glaucoma pathogenesis and may result in abnormal vasoconstriction and subsequent ONH and retinal ischaemic episodes in response to vasospastic stimuli^7^. Nicolela *et al*.^11^ noted that glaucoma patients had an abnormal increase in plasma endothelin-1 following body surface cooling, which could potentially lead to decreased ocular blood flow. However, in their study, as well as one by Gherghel *et al*.^5^, no significant changes were observed in retinal blood flow (measured by laser Doppler flowmetry) following body surface cooling. Similarly, Rojanapongpun *et al*.^29^ did not identify blood flow velocity changes in the ophthalmic artery following cold stimulation in eyes with POAG. However, Gherghel *et al*.^5^ reported a decrease in blood flow velocity, but not blood flow or volume, in the temporal neuroretinal rim following CPT in eyes with POAG. This change was not observed in healthy controls^5^. The current study did not find a significant change in ONH or macular VD in either healthy or glaucomatous eyes. This discrepancy between studies in haemodynamic changes following CPT may be related to variations in measurement methods, measured parameters, and examined vasculature. Of all the ocular haemodynamic evaluations performed to examine the effects of the CPT, a slight reduction in flow velocity at the temporal neuroretinal rim^5^ was the only change identified. This suggests that CPT-induced ocular haemodynamic changes are likely subtle and localized to specific vessels. Unfortunately, parameters that better represent haemodynamic changes (e.g., blood flow, volume, and velocity) cannot be directly measured using current OCT-A technology^5,30^. The OCT-A flow index provides information on flow velocity and is more sensitive than VD for detecting vasculature responses to hyperoxia^26^. Additionally, OCT transverse resolution is 15 µm, which is wider than capillary diameter (5–10 µm). As a result, VD measurements likely overestimate actual density and underestimate subtle VD changes^26^. Therefore, VD measured with OCT-A may not be sensitive enough to identify potential CPT-induced haemodynamic changes. Nevertheless, the CPT-induced VD changes observed in the current study were not simply caused by test-retest variability because baseline scans had high repeatability and CPT-induced changes were larger than scan-to-scan variations.

Another explanation for the controversial findings surrounding CPT-induced ocular haemodynamic changes may be that CPT only induces vasospasm in certain populations. Having cold extremities is a prominent feature of Flammer syndrome, which is primarily caused by vascular dysregulation and predisposes patients to vasospasm in response to cold or psychological stress^31^. A CPT-induced vasospasm in the digits is more common in patients with a positive history of cold extremities and is linked to ocular vasospasm and VF deficit worsening after CPT^12^. Therefore, we assumed that subjects with a self-reported history of “cold extremities” were also prone to cold-induced vasospasm. However, we did not find any significant differences in CPT-induced VD changes between subjects with and without a history of cold extremities in either healthy or glaucoma subjects. It may be that a positive subjective history of cold extremities is not a specific enough indicator for cold-induced nailfold capillary vasospasm. Vasospastic tendencies may be better identified with the addition of nailfold capillaroscopy. Doppler flowmetry measurements of ONH blood flow have been shown to be significantly related to finger blood flow in subjects with primary vascular dysregulation, as diagnosed using both history and nailfold capillaroscopy findings^32^.

The current study had several limitations. First, our study population was relatively small, which may have resulted in inadequate power to identify significant haemodynamic changes. We did identify a CPT-induced VD change that was greater than baseline VD measurement variability. Therefore, a study with a larger sample size may identify factors related to CPT-induced VD changes. Second, control subjects were younger than glaucoma subjects and did not have hypertension. These differences may have biased our comparison between baseline and post-CPT VD measurements. However, we did find that baseline VD was not correlated with age in normal controls, which is in agreement with a prior study that showed no association between subject age and macular VD^33^. Additionally, analyses that excluded hypertensive subjects did not change our overall results. Therefore, our findings were likely only minimally biased by age and hypertension differences between study groups. Third, although the classic CPT is conducted by immersion of one hand into ice water for 1–5 minutes^15,16^, other protocols with stronger stimuli have been used in the past in glaucoma patients, such as hand immersion in cold water for 15 minutes^12^, utilisation of a head-vest cooling garment for 30 minutes^11^, and the warm water test in combination with the CPT^5^. In the present study, we aimed to use a stress test that could be carried out in daily clinical practice; therefore, we opted for the 1-minute CPT. Further studies are needed to elucidate whether CPT with more intense stimuli or combined with warm water test will result in ocular haemodynamic changes that can be discerned by OCT-A. Lastly, this study only included glaucomatous eyes with retinal nerve fibre layer (RNFL) defects confined to one hemifield, in order to analyse whether the hemifields with and without RNFL defect would have different haemodynamic responses to stress. Glaucomatous eyes with advanced disease were excluded because these eyes would have very low baseline VD and the dynamic changes of VD might be limited and difficult to identify^25,27,34,35^. Thus, the results of this study can only be applied to subjects with early disease.

In conclusion, CPT did not induce significant changes in peripapillary or macular VD in healthy subjects or those with POAG. Additionally, OCT-A VD measurements may not be sensitive enough to evaluate stress-induced haemodynamic changes in the ONH and macula.

## Methods

### Study subjects

This study was approved by the Institutional Review Board of Taipei Veterans General Hospital (Taipei, Taiwan, approval ID: 2015-01-006BC). Informed consent was obtained from all participants, and the study was executed according to relevant guidelines and regulations. This study included 22 healthy controls and 23 patients with POAG. Subjects were between 30 and 65 years of age and all subjects were enrolled between March and December 2017. All included subjects had a best corrected visual acuity of 6/12 or better, a spherical equivalent refraction between −7 and +3 D, and an open angle (determined using gonioscopy). Subjects with a history of glaucoma filtering surgery, diabetic or hypertensive retinopathy, coronary artery disease, autoimmune disease, or cigarette smoking were excluded from the study. Patients with hypertension were included if BP was well controlled.

Subjects included in the control group had a normal IOP (≤21 mmHg), a normally appearing ONH and retina in both eyes. Patients were diagnosed with POAG by glaucoma specialists (C.J.L.L., Y.C.K., and M.J.C) if glaucomatous optic disc changes, corresponding RNFL loss, and reproducible VF defects were present. All POAG patients had well-controlled IOP with topical medications. Only eyes with RNFL defects confined to one hemifield on the Cirrus HD-OCT RNFL deviation map (Model 4000; Carl Zeiss Meditec, Inc., Dublin, CA, USA) were included. An RNFL defect was defined as the presence of 10 or more contiguous red or yellow superpixels that extended away from the optic disc margin corresponding to RNFL distribution.

All participants had undergone comprehensive ophthalmic examination within 3 months of enrollment, including autorefraction, best-corrected visual acuity assessment, axial length measurement (IOL Master 07740, Carl Zeiss Meditec AG, Jena, Germany), slit-lamp biomicroscopy, IOP measurement, gonioscopy, dilated fundus examination, fundus photography (digital retinal camera CX-1, Canon, Inc., Tokyo, Japan), and spectral-domain OCT (SD-OCT, Cirrus HD-OCT). Automated perimetry was also performed in glaucoma patients using the 24-2 Swedish interactive threshold algorithm standard algorithm of the Humphrey Field Analyser 750i (version 4.2, Zeiss-Humphrey Instruments, Dublin, CA, USA).

### Study design

Participants were instructed to avoid coffee and tea on the day of the CPT. Study measurements were performed between 1:00 and 5:00 pm in a dark room with a temperature of 20–25 °C. The PR, BP, and IOP were measured and a baseline OCT-A was performed without mydriasis. A subset of 18 subjects had two baseline scans to evaluate the repeatability of VD measurements. After baseline measurements were complete, the CPT was performed. This involved submerging the right hand in cold water (0–4 °C) up to the wrist for 1 minute. Immediately after the CPT, another OCT-A measurement was performed, followed by post-CPT PR and BP measurements.

All OCT-A scans were performed using the AngioVue system (version 2016.1.0.23) incorporated into the RTVue XR Avanti SD-OCT (Optovue, Inc. Fremont, CA, USA). This system uses the split-spectrum amplitude-decorrelation angiography algorithm to detect red blood cell movement within retinal and choroidal blood vessels to generate high-resolution *en face* images of the perfused vasculature^36^. The VD was then calculated as the percentage of the area occupied by perfused blood vessels in the scanned image.

The ONH scan covered an area of 4.5 × 4.5 mm and was centred on the optic disc. The peripapillary VD was measured using blood vessel signals present between the internal limiting membrane and the posterior RNFL boundary. The macular scan had an area of 3 × 3 mm and was centred on the fovea. Superficial macular VD measurements were made by extracting signals between the internal limiting membrane and the posterior inner plexiform layer boundary. All measurements were performed by the same technician to reduce inter-operator variability. Additionally, OCT-A images were reviewed to ensure that only images with appropriate segmentation and an adequate signal strength index (≥35) were included in analyses.

**Figure 1:**
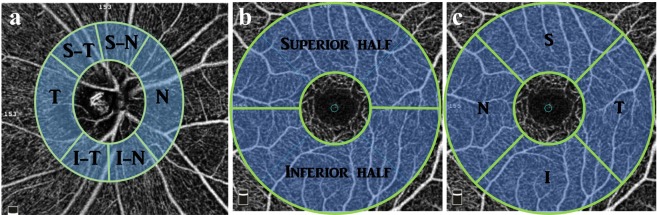
Optical coherence tomography angiography images of the superficial retinal vascular plexus in the peripapillary (**a**) and parafoveal (**b**,**c**) regions. Measurement sectors for each region are also shown. S-T, superior-temporal; S-N, superior-nasal; I-T, inferior-temporal; I-N, inferior-nasal; I, inferior; S, superior; N, nasal; T, temporal.

The AngioVue software provided whole image and sectoral measurements. In the ONH scan, peripapillary VD was measured within a 0.75 mm wide elliptical annulus that extended from the optic disc boundary and was divided into the following six sectors: nasal (N), superior nasal (S-N), superior temporal (S-T), temporal (T), inferior temporal (I-T), and inferior nasal (I-N, Fig. 1a). In the macular scan, the parafoveal region was measured within an annulus that had an inner diameter of 1 mm and an outer diameter of 3 mm. The annulus was divided into superior and inferior halves (Fig. 1b) for some analyses and quartered into temporal (T), superior (S), nasal (N), and inferior (I) quadrants for others (Fig. 1c).

The superior and inferior hemifields of glaucomatous eyes were classified as damaged or undamaged according to the RNFL defect location (as discussed above) to evaluate the potential impact of disease severity on stress-induced haemodynamic changes. Representative sectoral VD measurements were selected so that the superior hemifield was represented by the S-T and S-N sectors in the peripapillary scan and the S quadrant and superior half in the macular scan. Inferior hemifield measurements were obtained in the same way using the I-T and I-N sectors in the peripapillary scan and the I quadrant and inferior half in the macular scan.

### Statistical analysis

One eye of each subject was selected for analysis. The eye with the higher OCT-A signal strength index was selected if both eyes were eligible. Repeatability of OCT-A measurements was evaluated using the ICC, with ICC estimates and 95% CI calculated using a mean-rating, absolute-agreement, 2-way mixed-effects model. The CV was calculated for repeated baseline measurements and OCT measurement variation in response to CPT. Nonparametric tests were used for the following comparisons because most measurement data were not normally distributed. For subjects with two baseline scans, the mean was used as the baseline value. The impact of CPT on VD was first evaluated by comparing baseline and post-CPT VD measurements using the Wilcoxon signed rank test. The CPT-induced change in VD was calculated by subtracting the baseline value from the post-CPT value. Measurements were compared between healthy and POAG subjects and between subjects with and without a history of cold extremities using Mann-Whitney *U* tests. Post-CPT VD changes among healthy eyes, the undamaged and damaged hemifields in glaucomatous eyes were compared using Kruskal-Wallis test. Categorical variables were analysed using chi-square tests. Statistical significance was defined as P ≤ 0.05 and all analyses were conducted using SPSS statistical software (version 22, SPSS Inc., Chicago, IL, USA).

### Ethics approval

The study was reviewed and approved by the Institutional Review Board of Taipei Veterans General Hospital.

## Data Availability

Datasets from the current study are not publicly available due to compliance to privacy. Summary statistics are available from the corresponding author on reasonable request.
